# On the Social Construction of Overdiagnosis: Comment on "Medicalisation and Overdiagnosis: What Society Does to Medicine"

**DOI:** 10.15171/ijhpm.2017.21

**Published:** 2017-02-25

**Authors:** Bjørn Hofmann

**Affiliations:** ^1^ Department for the Health Sciences, Norwegian University of Science and Technology (NTNU), Gjøvik, Norway.; ^2^ The Centre for Medical Ethics, University of Oslo, Oslo, Norway.

**Keywords:** Overdiagnosis, False Test Results, Social Construction, Medicalization

## Abstract

In an interesting article Wieteke van Dijk and colleagues argue that societal developments and values influence the practice of medicine, and thus can result in both medicalisation and overdiagnosis. They provide a convincing argument that overdiagnosis emerges in a social context and that it has socially constructed implications. However, they fail to show that overdiagnosis per se is socially constructed and how this construction occurs. Moreover, the authors discuss overdiagnosis on a micro level without acknowledging that overdiagnosis cannot be observed in individuals "in the doctor’s office." We cannot tell whether a diagnosed person is overdiagnosed or not. This is the core of the problem. Despite these shortcomings, Wieteke van Dijk and her colleagues are certainly on to something important, and they should be encouraged to elaborate their perspective. We certainly need to deepen our understanding of the social construction of overdiagnosis.

In an interesting and important article Wieteke van Dijk and colleagues argue that societal developments and values influence the practice of medicine, and thus can result in both medicalisation and overdiagnosis.^1^ They point out that while the social aspects of medicalization are acknowledged, more emphasis should be placed on the societal aspects of overdiagnosis.

To make their point they underscore the value-ladenness of disease. Where we decide set our cut-off values (for hypertension for example) depends on risk assessments, and ultimately on our norms and values. Furthermore, focusing on specific risk factors (such as hypertension) may make us ignore a wide range of other important factors, such as life style and socioeconomic context, they argue. Our social propensity to look for quick fixes deflects our focus from important aspects. Hence, our social norms and values make us receptive for overdiagnosis.

To illustrate how social aspects influence both medicalization and overdiagnosis they use three examples: care for the mentally disabled, treatment for Alzheimer disease, and medicalization of childbirth. The examples underscore how indication thresholds and diseases are socially constructed.

Van Dijk and colleagues conclude that “[m]edicalisation and overdiagnosis hold an ambivalent relationship.” Their main point is that (*a*) medicalization “follows from overdiagnosis in the doctor’s office” and that (*b*) overdiagnosis follows from societal norms and values (facilitating medicalization).

Although van Dijk and colleagues are very close to my own conceptions of both overdiagnosis and medicalization,^2-5^ I think their perspective deserves some further reflection. Allow me to consider their last claim first, ie, that overdiagnosis is the implication of societal norms and values. This is a fairly trivial statement and very few would disagree. The authors fail to make it clear to what extent overdiagnosis as such, and not only its implications, is a social construction. Pointing to the fact that indication thresholds and cut-off values are derived from social norms and values does not do the trick, because these thresholds decide the level of uncertainty we are willing to accept, ie, how we trade off false positive against false negative test results. For example, a radiologist may be more afraid of missing pathology (and accepting more false positives) than the referring general practitioner (GP), who on her side may be more focused on a conclusive diagnosis (and accepting more false negatives). However, it is important to notice that the issue with thresholds and cut-off values is the social (or professional) acceptance of *false* results. However, overdiagnosis is about *correct* test results.

As the authors rightly point out, overdiagnosis is defined as the detection of abnormalities that will never cause symptoms or death. The abnormalities that are overdiagnosed are correctly identified. They are true positives.^6^ Hence, overdiagnosis is not about how we trade off two types of test error, it is about how we handle a specific type of uncertainty, ie, the uncertainty of whether a given condition (“abnormality”) will cause symptoms, disease, or death. Certainly, it is about risk aversion. If we are very much afraid of a certain disease, we will go great lengths to detect and defeat it^7^ and we will accept harms, such as overdiagnosis, to do so. Undoubtedly, our risk aversion is socially constructed, and our eagerness to find conditions that may result in disease is socially constructed. Surely, this explains how we come to accept overdiagnosis and its extension, but it does not explain how overdiagnosis as such is socially constructed. Hence, we still need van Dijk and colleagues to help us explain how exactly overdiagnosis is socially constructed.

Allow me now to turn to the authors’ first insight, ie, that medicalization “follows from overdiagnosis in the doctor’s office.” Here it may be fruitful to pay attention to two important aspects of overdiagnosis. First, that overdiagnosis does not appear in the doctor’s office. In her office, the doctor only sees the patient and the (true) positive test result. She does not know whether the patient will develop symptoms or disease or die as a consequence of having the detected condition. And when treating the patient for the identified condition, the doctor will never know whether the patient in her office is overdiagnosed. The patient will never know either. And both will happily think that a life has been saved. This is called “the popularity paradox.”^8,9^ Therefore, you have to be prophetic to observe overdiagnosis in the individual^2^ (“in the doctor’s office”) and overdiagnosis can only be measured indirectly on a populational level^10^ or as a pathological reserve.^8,11^

Second, the authors are correct in underscoring that both “overdiagnosis and medicalisation result in more people receiving a medical diagnosis.” However, overdiagnosis does not result in medicalization. Overdiagnosis does not medicalize a condition, because it is already medical.^5^ Ductal carcinoma in situ (DCIS) and colorectal polyps are already identified as medically relevant conditions, ie, conditions that potentially can result in harmful or deadly disease, ie, breast cancer and colorectal cancer respectively. Again, we may very well dwell with the social conditions that make these conditions (DCIS and polyps) medically relevant and make us screen for them. However, this does not make the persons with the overdiagnosed conditions medicalized.

Of course, the same norms and values may be identified in medicalization as in the process of overdiagnosis. They may even have the same drivers^12^ and mechanisms, such as “the increasing societal consciousness of conditions and its treatments, decreasing the individual and societal tolerance to endure everyday complaints.”^1^ However, this is different than saying that overdiagnosis is socially constructed.

My concerns may of course be based on my misreading of van Dijk and colleagues, and if this is that case I sincerely apologize. But if I have understood them well and even if my critique is relevant, I do think that they have a good case. Overdiagnosis is an obvious candidate for being a social construction. My point is only that more work is needed. Wieteke van Dijk and colleagues’ contributions to this work are most welcome and needed.

## Ethical issues

Not applicable.

## Competing interests

Author declares that he has no competing interests.

## Author’s contribution

BH is the single author of the paper.
